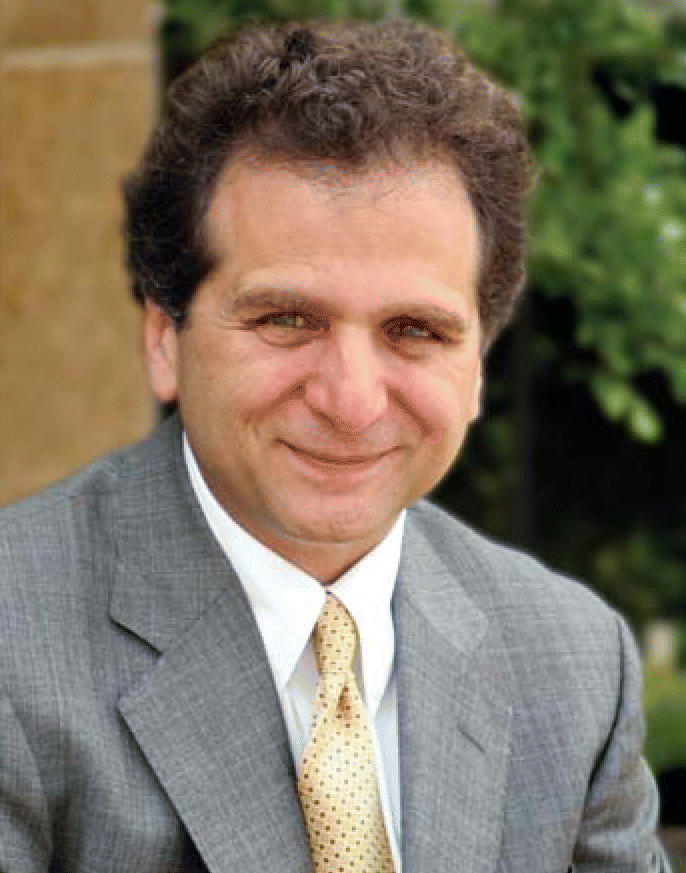# A Year in Review

**Published:** 2006-09

**Authors:** David A. Schwartz

**Affiliations:** Director, NIEHS and NTP, E-mail: david.schwartz@niehs.nih.gov

A little more than a year ago, I joined the NIEHS as director. As I reflect on this past year, I am proud of the many accomplishments I and the staff of the NIEHS have worked together to achieve, particularly our strategic plan, *New Frontiers in Environmental Sciences and Human Health* (http://www.niehs.nih.gov/external/plan2006/home.htm), which highlights our future challenges and goals. I am confident that we will continue to advance this bold plan for the institute and achieve a profound impact on the understanding of human biology and health.

Many of the goals of the new strategic plan are already coming to fruition. I am pleased that we have launched the DISCOVER (Disease Investigation through Specialized Clinically-Oriented Ventures in Environmental Research) Program and the Director’s Challenge, both of which focus on the interface between basic biological mechanisms and clinical research to unravel the complexity of environmentally influenced diseases. Our investment in these programs of $11 million in fiscal year 2007 exemplifies our commitment to this important area of research and to alleviating the substantial health burden such diseases pose to society.

Recently, we received considerable endorsement from the scientific community, Congress, and the federal administration for the Genes and Environment Initiative (GEI), a bold new trans-NIH venture to accelerate research discoveries on the role of genes and the environment in human disease. The NIEHS is taking a lead role in this initiative through the GEI Exposure Biology Program, which will promote the development and application of new technologies to precisely measure human exposure in population genomics studies. This program builds on the unique strengths and leadership in environmental health science gained through the research programs of the NIEHS and the National Toxicology Program. In May 2006, the NIEHS hosted the Exposure Biology Workshop to lay the groundwork for this innovative program. The workshop convened individuals with diverse backgrounds in toxicology, biology, genetics, public health, epidemiology, nanotechnology, engineering, physics, and informatics to identify research opportunities that may inform population genomics studies in an immediate and profound way. The Environmental Airway Disease Project, which focuses on biomarkers in the lung that are triggered by environmental agents, was launched in fiscal year 2006 as the initial research project under this program.

I am particularly proud of the progress we have made in fostering clinical and translational research and training at the NIEHS. This is an exciting area of emphasis for the NIEHS, uniquely positioning us to take advantage of opportunities to put into practice the basic research discoveries of our scientists in environmental health sciences. William Martin, a nationally recognized physician-scientist, has joined the NIEHS to head the new Office of Translational Research. The focus of this office will be to accelerate the application of basic research discoveries to patient treatment and disease prevention, both nationally and internationally. In addition, our new Clinical Research Unit, directed by Perry Blackshear, is fast becoming a reality; we hope to have an 8,000-square-foot facility on the NIEHS campus by the spring of 2007. This new facility will allow us to fully integrate into our intramural research program a strong clinical research program focused on the biology of complex human diseases.

The environmental health science field is uniquely poised to benefit from the incredible pace of biomedical discovery that has resulted from new advances in genetics, molecular biology, computational sciences, and the physical sciences. The NIEHS is working hard to meet this challenge through targeted investments in training the next generation of environmental health scientists. Recently, we launched two programs called ONES (Outstanding New Environmental Scientists) and STEER (Short Term Educational Experiences for Research) to attract and support the best and brightest new researchers to our field. I am pleased to announce that the first eight recipients of the ONES awards have already been selected. The NIEHS also continues to provide a positive work environment for research and training of postdocs; this year we were again voted among the top five places for postdocs to work in the country. This exemplifies our commitment to supporting the future leaders in environmental health science.

NIEHS-funded scientists continue to unravel important biomedical problems. In the past year, we have made many significant breakthroughs, including

- the identification of predisposing genetic mutations for breast cancer that may improve the detection and treatment of disease in high-risk families and women;
- the discovery of the role of endogenous airway relaxants that may be targeted in the treatment of chronic airway hyperresponsiveness and chronic airway inflammation;
- the elucidation of antiinflammatory effects of glucocorticoids that may be targeted in therapeutic regimens for environmental diseases such as asthma, autoimmune disease, and sepsis;
- the discovery of mechanistic links between inhaled particulate matter in urban areas and susceptibility to cardiovascular disease;
- the elucidation of regulatory mechanisms for synaptic plasticity in the brain that impact learning and memory; and
- the identification of the structural basis for errors in DNA synthesis due to strand misalignment, which may result from environmental stress and have profound impacts on human disease.

We continue in our commitment to improving the health of residents impacted by Hurricane Katrina in the U.S. Gulf Coast region. We have created a public–private partnership to support a new program called HEAL (Head off Environmental Asthma in Louisiana). Modeled after the Inner-City Asthma Study, the program’s goal is to assess the problem of childhood asthma resulting from exposure to excessive amounts of mold, microbial toxins, and airway pollutants, and ultimately to find ways to address the illness. This program will be the foundation for a new Global Health Initiative to address pressing environmental health problems worldwide.

I am extremely proud of the progress we have made in the past year, and I am confident that if we remain focused on our goals and follow the plan we’ve established, we will continue to achieve great things together. I fully appreciate your support and advice.

## Figures and Tables

**Figure:**